# Recombinant BCG Expressing HTI Prime and Recombinant ChAdOx1 Boost Is Safe and Elicits HIV-1-Specific T-Cell Responses in BALB/c Mice

**DOI:** 10.3390/vaccines7030078

**Published:** 2019-08-02

**Authors:** Athina Kilpeläinen, Narcís Saubi, Núria Guitart, Alex Olvera, Tomáš Hanke, Christian Brander, Joan Joseph

**Affiliations:** 1 Catalan Center for HIV Vaccine Research and Development, AIDS Research Unit, Infectious Diseases Department, Hospital Clínic/IDIBAPS, 08036 Barcelona, Catalonia, Spain; 2 Vall d’Hebron Research Institute, Hospital Universitari Vall d’Hebron, 08035 Barcelona, Catalonia, Spain; 3 Irsicaixa AIDS Research Institute, 08916 Badalona, Catalonia, Spain; 4 Universitat de Vic-Universitat Central de Catalunya (UVic-UCC), 08500 Vic, Barcelona, Spain; 5 Nuffield Department of Medicine, The Jenner Institute, University of Oxford, Oxford OX3 7DQ, UK; 6 International Research Center of Medical Sciences (IRCMS), Kumamoto University, Kumamoto 860-0811, Japan; 7 ICREA, Pg. Lluís Companys 23, 08010 Barcelona, Catalonia, Spain; 8 AELIX Therapeutics, 08028 Barcelona, Catalonia, Spain

**Keywords:** BCG, HIV, vaccine, rBCG, HTI, T-cell

## Abstract

Despite the availability of anti-retroviral therapy, HIV-1 infection remains a massive burden on healthcare systems. Bacillus Calmette-Guérin (BCG), the only licensed vaccine against tuberculosis, confers protection against meningitis and miliary tuberculosis in infants. Recombinant BCG has been used as a vaccine vehicle to express both HIV-1 and Simian Immunodeficiemcy Virus (SIV) immunogens. In this study, we constructed an integrative *E. coli*-mycobacterial shuttle plasmid, p2auxo.HTI.int, expressing the HIVACAT T-cell immunogen (HTI). The plasmid was transformed into a lysine auxotrophic *Mycobacterium bovis* BCG strain (BCG*ΔLys*) to generate the vaccine BCG.HTI^2auxo.int^. The DNA sequence coding for the HTI immunogen and HTI protein expression were confirmed, and working vaccine stocks were genetically and phenotypically characterized. We demonstrated that the vaccine was stable in vitro for 35 bacterial generations, and that when delivered in combination with chimpanzee adenovirus (ChAd)Ox1.HTI in adult BALB/c mice, it was well tolerated and induced HIV-1-specific T-cell responses. Specifically, priming with BCG.HTI^2auxo.int^ doubled the magnitude of the T-cell response in comparison with ChAdOx1.HTI alone while maintaining its breadth. The use of integrative expression vectors and novel HIV-1 immunogens can aid in improving mycobacterial vaccine stability as well as specific immunogenicity. This vaccine candidate may be a useful tool in the development of an effective vaccine platform for priming protective responses against HIV-1/TB and other prevalent pediatric pathogens.

## 1. Introduction

According to the latest reports, there are currently 37 million people infected with HIV, the majority of whom live in sub-Saharan Africa [1]. Despite the existence of anti-retroviral therapy, 1.8 million people were newly infected in 2017, and almost one million people died due to HIV-related disease [1]. Developing a safe, efficacious, and accessible HIV vaccine would be the optimal solution for the prevention of HIV-1 infection as well as reduction of HIV-related diseases. Evidence demonstrating the role of T-cell responses directed against HIV-1 in the control of viral replication is growing [2,3]. HIV-1-specific CD8^+^ T-cells have been detected in exposed seronegative individuals, and CD8^+^ cytotoxic T-lymphocytes (CTL) responses targeting HIV-1 Gag have been associated with reduced viral loads in infected individuals [4,5,6]. 

The “HIVACAT T-cell immunogen” (HTI) was designed to cover T-cell targets, against which T-cell responses are predominantly observed in HIV-1-infected individuals with low HIV-1 viral loads [7]. This immunogen has been manufactured as naked DNA, as well as introduced into viral vaccine vectors and an immunization regimen consisting of three injections of DNA encoding HTI followed by a boost with modified vaccinia Ankara (MVA)-vectored HTI has been shown in C57BL/6 mice to result in the induction of HIV-1 specific T-cell responses to most antigen regions included in its design. High magnitudes of HIV-1 specific T-cells were also induced in macaques following three injections of DNA.HTI and two injections of MVA.HTI [7]. It is well known that delivering a boosting injection of an immunogen using a viral vector such as MVA can boost the response of DNA immunization and increase the magnitude of responses. Such heterologous prime-boost regimens have been used for a number of immunogens in the context of HIV [8]. 

Upon intramuscular delivery, DNA vaccines are thought to induce cellular immunity through antigen synthesis and presentation to T-cells. The immunity is induced by the direct transfection of antigen-presenting cells (APCs) or through cross-presentation by APCs [9,10]. Similar to DNA, the *Mycobacterium bovis* strain, Bacillus Calmette-Guérin (BCG), when used as a vaccine vector also induces immunity by targeting APCs, although this occurs not through transfection, but rather through infection. Recombinant BCG is a promising live attenuated bacterial vaccine vector for inducing T-cell immunity, as it is a slow-growing organism that provides a persistent low-level antigenic exposure upon the infection of macrophages and APCs, and could drive effector and memory T-cell responses [11]. In the context of anti-tuberculosis (anti-TB) immunity, the pathogen for which BCG currently is licensed as a vaccine, immune responses in humans are predominantly T helper 1 (Th1)-cell mediated. Infected dendritic cells migrate to lymph nodes and activate antigen-specific CD4^+^ T-cells in the presence of cytokines such as interleukin (IL)-18 and IL-12 [12]. The T-cell repertoire induced by vaccination is broad, targeting multiple mycobacterial antigens [13]. BCG is safe and is currently administered to 80% of infants in countries where it is part of the national childhood immunization program [14]. Aside from the induction of anti-TB immune responses, vaccination has been linked to decreased mortality due to other infections in infants [15,16]. This phenomenon has been related to the enhanced monocyte function observed in humans following vaccination, which is characterized by CD11b, TLR-4 expression, and increased cytokine production [17].

Recombinant BCG (rBCG) has been utilized as a vector to express HIV-1/Simian Immunodeficiency Virus (SIV) antigens and assessed regarding the induction of specific T-cell responses in several animal models [18]. The need for adjuvants is overcome as its cell-wall peptidoglycans and lipoproteins act as an adjuvant on their own [19,20,21,22]. BCG also has several advantages as a live vaccine vehicle, it is easy to mass-produce with low cost, and is heat stable [23]. It is also suitable for neonates, as vaccination is not affected by maternal antibodies [24,25]. Finally, it has a proven safety record after having been delivered as a TB vaccine to over three billion individuals [26]. As with DNA vaccination, rBCG-based HIV vaccines on their own may induce low-level specific immune responses, and thus they are often combined with virally vectored immunogens in heterologous prime-boost vaccination regimens where they have been shown to increase HIV-1 specific immune responses [27,28,29,30]. However, a rBCG expressing HIV-1 group M consensus Env vaccine on its own was shown to induce comparable immune responses both in the female reproductive tract and lungs when compared with adenovirus prime/recombinant vaccinia virus boost immunization [31]. The applicability of rBCG as a priming agent was demonstrated by Ami *et al.*, where priming with rBCG and boosting with a replication-deficient vaccinia virus strain expressing SIVgag was able to confer protection in Cynomolgus macaques against mucosal challenge with pathogenic SHIV. Interestingly, effective protection was not achieved in animals receiving the opposite combination or the vaccine modalities delivered on their own [32]. These data support the use of rBCG as a priming vector for an HIV-1 vaccine, whereby the use of rBCG could potentially strengthen or qualitatively modify the immune response when combined with DNA and/or a viral vector expressing an HIV-1 immunogen.

Here, we present the construction and characterization of recombinant BCG expressing the HTI immunogen, the BCG.HTI^2auxo.int^ vaccine, harboring the integrated 2auxo expression cassette in their chromosome. The HTI immunogen has previously been assessed in mice and macaques when delivered as a prime-boost regimen vectored by DNA and MVA [7]. We demonstrate that BCG.HTI^2auxo.int^ delivered in combination with ChAdOx1.HTI increases the HIV-1 specific T-cell responses in adult BALB/c mice. Priming with wild type BCG (BCGwt) was also shown to similarly increase the magnitude of the response, but significantly decreased the breadth of the T-cell responses. Furthermore, we demonstrate that priming with rBCG, in some mice, can alter the immunodominance profile of the vaccine-induced T-cell response. 

## 2. Materials and Methods 

### 2.1. Construction of the BCG.HTI^2auxo.int^ Strain Using an Antibiotic-Free Plasmid Selection System 

The double auxotrophic *E. coli*–mycobacterial shuttle integrative vector, the p2auxo.int plasmid, was previously constructed in our laboratory [33]. This vector contains the *glyA* and *LysA* genes, which function as an antibiotic-free selection and maintenance system in the auxotrophic strains of *E. coli* M15*ΔglyA* and BCG*ΔLys*, respectively. It also contains sites (*attP*) for integration into the BCG genome at the *attB* site. The synthetic sequence of HTI [7] was codon-optimized for BCG expression to match the G+C rich mycobacterial codon usage for enhanced expression [34]. The HTI G+C rich DNA sequence was synthesized by Geneart (USA) and ligated to the integrative p2auxo.int plasmid fused to the 19-kDa lipoprotein secretion signal sequence generating p2auxo.HTI^int^. The ligation products were subsequently transformed into the *E. coli* M15*ΔglyA* strain for growth and selection.

### 2.2. Bacterial Cultures and Transformation

Cells of the glycine auxotrophic strain of *E. coli,* M15*ΔGly*, provided by Dr. Pau Ferrer (Universitat Autònoma de Barcelona, Spain), were cultured in LB supplemented with glycine (70 μg/mL). The *E. coli* M15*ΔGly* cells were transformed with the p2auxo.HTI^int^ plasmid by electroporation. For this, the *E. coli* cultures were grown to an optical density of 0.125 at 600 nm, as well as concentrated and transformed using a Bio-Rad gene pulser electroporator at 2.5 kV, 25 μF, and 200 Ω. The transformed cells were subsequently cultured on M9-D agar plates (minimal M9-derivative medium: Na_2_HPO_4_, 6.78 g/L; KH_2_PO_4_, 3 g/L; NaCl, 0.5 g/L; NH_4_Cl, 1 g/L, glucose, 10 g/L; MgSO_4_, 2 mmol/L; CaCl_2_, 0.1 mmol/L; thiamine, 0.1 g/L; FeCl_3_, 0.025 g/L; AlCl_3_·6H_2_O, 0.13 mg/L; ZnSO_4_·7H_2_O, 2.6 mg/L; CoCl_2_·6H_2_O, 0.47 mg/L; CuSO_4_·H_2_O, 4.6 mg/L; H_3_BO_3_, 0.03 mg/L; MnCl_2_·4H_2_O, 4.2 mg/L; NiCl_2_·6H_2_O, 0.02 mg/L; Na_2_MoO_4_·2H_2_O, 0.06 mg/L, with 1.5% bactoagar added) without glycine supplementation for selection or with glycine supplementation as a control. The QIAprep Spin Miniprep Kit was used according to the manufacturer’s instructions (Qiagen, Hilden, Germany) to extract plasmid DNA from *E. coli*. The resulting plasmids were tested for identity and correct insertion by PCR and restriction enzyme profiling. The selected plasmid was transformed into BCG*Δlys*.

The lysine auxotrophic BCG strain, BCG*Δlys*, kindly provided by W.R. Jacobs Jr., B.R. Bloom, and T. Hsu (Albert Einstein College of Medicine, New York, NY, USA), was transformed with p2auxo.HTI^int^ plasmid by electroporation. The mycobacteria were cultured in Middlebrook 7H9 broth medium or on Middlebrook agar 7H10 medium supplemented with albumin–dextrose–catalase (ADC; Difco Laboratories, Franklin Lakes, NJ, USA) containing 0.05% Tween 80. L-lysine monohydrochloride (Sigma) was dissolved in distilled water and used as a supplement at a final concentration of 40 μg/mL. For transformation, BCG was cultured to an optical density of 1.5 at 600 nm, washed with 10% glycerol, concentrated, and transformed using a Bio-Rad gene pulser electroporator at 2.5 kV, 25 μF, and 1000 Ω. Then, the transformants were cultured on ADC-supplemented Middlebrook agar 7H10 medium containing 0.05% Tween 80 without lysine supplementation. The resulting colonies were assessed for plasmid insertion, integrity, and HTI expression. From a selected colony, a Master Seed (MS) and a Working Vaccine Stock (WVS) were produced according to the seed lot system. For the BCG substrain identification assay, the commercial BCG Danish 1331 strain (Pfizer, New York, NY, USA) kindly provided by Dr. Neus Altet (Urology Department at Hospital Clínic de Barcelona, Barcelona, Spain), and the commercial BCG Connaught strain (ImmuCyst, Aventis, Paris, France) were used as standards.

### 2.3. Sodium Dodecyl Sulfate–Polyacrylamide Gel Electrophoresis and Western Blot Analysis

Cell lysates of mid-logarithmic phase BCG transformants were prepared by sonication in a protein extraction buffer (50 mmol/L Tris–HCl pH 7.5, 5 mmol/L Ethylenediaminetetraacetic acid (EDTA), 0.6% sodium dodecyl sulfate) containing protease inhibitor cocktail (Sigma-Aldrich, St Louis, MO, USA). Cell lysates supernatants were subsequently separated by a Novex 4–12% Bis-Tris SDS-PAGE gel (Thermo Fisher Scientific, Waltham, MA, USA), and electroblotted onto a pretreated polyvinylidene difluoride membrane using an iBlot kit (Thermo Fisher Scientific, Waltham, MA, USA). The HTI protein was stained using the primary anti-HTI monoclonal antibodies n63 and n69 at 5 µg/mL overnight kindly provided by Aelix Therapeutics (Barcelona) followed by secondary goat anti-mouse Immunoglobulin G-Horse Radish Peroxidase(IgG–HRP) antibody (Jackson ImmunoResearch, Cambridgeshire, UK) for 1 h diluted at 1:10,000. The membrane was developed using the SuperSignal™ West Femto Maximum Sensitivity Substrate kit (Thermo Fisher Scientific, Waltham, MA, USA).

### 2.4. In Vitro Stability of the BCG.HTI^int^ Strain

Five subcultures (∼35 bacterial generations) from the MS of BCG.HTI^2auxo.int^ harboring the p2auxo.HTI^int^ plasmid DNA (two selected clones) containing the lysine complementing gene were cultured in 7H9 Middlebrook broth with and without L-lysine selection. Subcultures were performed every 7 days by transferring 100 µL of the stationary phase culture to 5 mL of fresh medium. PCR analysis of the HTI DNA coding sequence were performed using insert specific primers designed at both HTI 3’ and 5’ sequences. PCR product size from the p2auxo.HTI.int plasmid and subcultures were compared. 

### 2.5. Mycobacterial Genomic DNA Preparation for the Multiplex PCR Assay and for attR and attL DNA Regions PCR

For isolation of DNA from BCGwt, BCG.HTI^2auxo.int^; 2 mL of mycobacterial culture was centrifuged at 5000 rpm for 10 min at room temperature. The pellet was resuspended in 200 μL of distilled water and heated at 95 °C for 20 min to inactivate and lyse bacterial cells. The sample was next centrifuged at a speed of 13,000× *g*. A total of 5 μL of supernatant was used for the amplification reaction. The commercial BCG strains were treated similarly, except in this case, 400 µL of the reconstituted freeze-dried flasks were used.

### 2.6. Multiplex PCR Assay for M. bovis BCG Substrain Pasteur Identification

The multiplex PCR assay was performed as described previously by Bedwell *et al.* [35], using 5 µL of mycobacterial DNA isolated from BCG.HTI^2auxo.int^, BCG wt (Pasteur strain, kindly provided by W.R. Jacobs Jr., B.R. Bloom, and T. Hsu (Albert Einstein College of Medicine, New York, NY, USA)), and commercial BCG strains as template in a final reaction volume of 50 µL. 

### 2.7. Immunization of Mice and Isolation of Splenocytes 

Groups of eight adult (seven-week-old) female BALB/c mice were immunized intradermally in one footpad, and two groups were left unimmunized. The first group received 10^5^ colony-forming units (CFU) of BCG.HTI^2auxo.int^ (Group A), the second group received 10^6^ CFU of BCG wt (Group B), both groups in one footpad. Two groups were left unimmunized (Groups C and D). ChAdOx1.HTI was constructed as previously described [36], and groups A–C were boosted intramuscularly with 10^9^ viral particles (vp) after five weeks, while group D was left unimmunized. All the mice were sacrificed two weeks after the boost for immunogenicity analyses. Immediately following sacrifice of the animals, splenocytes were harvested and homogenized using 70 µm cell strainers (Falcon; Becton Dickinson, Franklin Lakes, NJ, United States) and 5-ml syringe rubber plungers. Red blood cells were removed with ACK lysing buffer (Lonza, Barcelona, Spain), and the splenocytes were washed and resuspended in complete medium (R10 (RPMI 1640 supplemented with 10% fetal calf serum and penicillin–streptomycin), 20 mmol/L of HEPES, and 15 mmol/L of 2-mercaptoethanol).

### 2.8. IFN-γ ELISpot Analysis

The Enzyme-linked immune absorbent spot (ELISpot) assay was performed using the commercial murine IFN-γ ELISpot kit (Mabtech, Nacka Strand, Sweden) according to the manufacturer’s instructions. The ELISpot plates (MSISP4510, 96-well plates with polyvinylidene difluoride membranes, Millipore, USA) were 70% EtOH treated and coated with purified anti-mouse interferon-γ (IFN-γ) capture monoclonal antibody diluted in phosphate-buffered saline (PBS) to a final concentration of 5 µg/mL at 4 °C overnight. Then, 250,000 fresh splenocytes were added to each well and stimulated with 17 peptide pools containing a total of 147 15 mer overlapping peptides (OLP) spanning the HTI sequence, at a concentration of 10 μg/mL per peptide. Tuberculin purified protein derivative (PPD, AJ vaccines, Copenhagen, Denmark,) at a concentration of 5 μg/mL was used to assess TB-specific responses. All the samples and controls were plated in duplicate wells. ELISpot assays were incubated for 16 h at 37 °C, 5% CO_2_. The plates were subsequently washed 5× with PBS, incubated for 2 h with a biotinylated anti-IFN-γ monoclonal antibody (mAb) diluted in PBS 2% Fetal Calf Serum (FCS) to a final concentration of 2 µg/mL, washed 5× in PBS, and incubated with the streptavidin–alkaline phosphatase conjugate in PBS 2% FCS. Then, plates were washed 5× with PBS before incubating with 100 µL of 5-bromo-4-chloro-3-indolyl phosphate (BCIP)/nitro blue tetrazolium (NBT) substrate solution (Sigma-Aldrich, St Louis, MO, USA). After 5–10 min, the plates were washed with tap water, dried, and the resulting spots counted using an ELISPOT reader (Autoimmune Diagnostika GmbH, Strassberg, Germany). For each animal, the mean of background responses was subtracted individually from all the wells to enable a comparison of the IFN-γ spot forming cells (SFC)/10^6^ between groups. To define positive responses, a threshold was defined as at least five spots per well, and responses exceeding the mean number of spots in negative control wells plus three standard deviations of the negative control wells.

### 2.9. Statistical Analysis 

Immunogenicity data is represented as group means of the total IFN-γ SFC/10^6^ response or as medians for individual antigens/pools. Statistical differences were assessed by ordinary one-way analysis of variance when comparing total ELISpot responses or the Kruskal–Wallis test when comparing responses to individual pools. (* *p* < 0.05; ** *p* < 0.01; *** *p* < 0.001). GraphPad Prism 6.0 (Graphpad, San Diego, CA, USA) software was used. Body mass data are shown as group means with error bars indicating standard deviation as well as means ± 2 standard deviation (SD) from naïve mice. Statistical analyses were performed using the Kruskal–Wallis test. 

### 2.10. Ethics Statement 

The animal experiments strictly conformed to the animal welfare legislation of the Generalitat de Catalunya. All the experiments were approved by the local Research Ethics Committee (Procedure Med 365/16, Clinical Medicine, School of Medicine, University of Barcelona). 

## 3. Results

### 3.1. Construction of the BCG.HTI^2auxo.int^ Vaccine Strain

**Figure 1:**
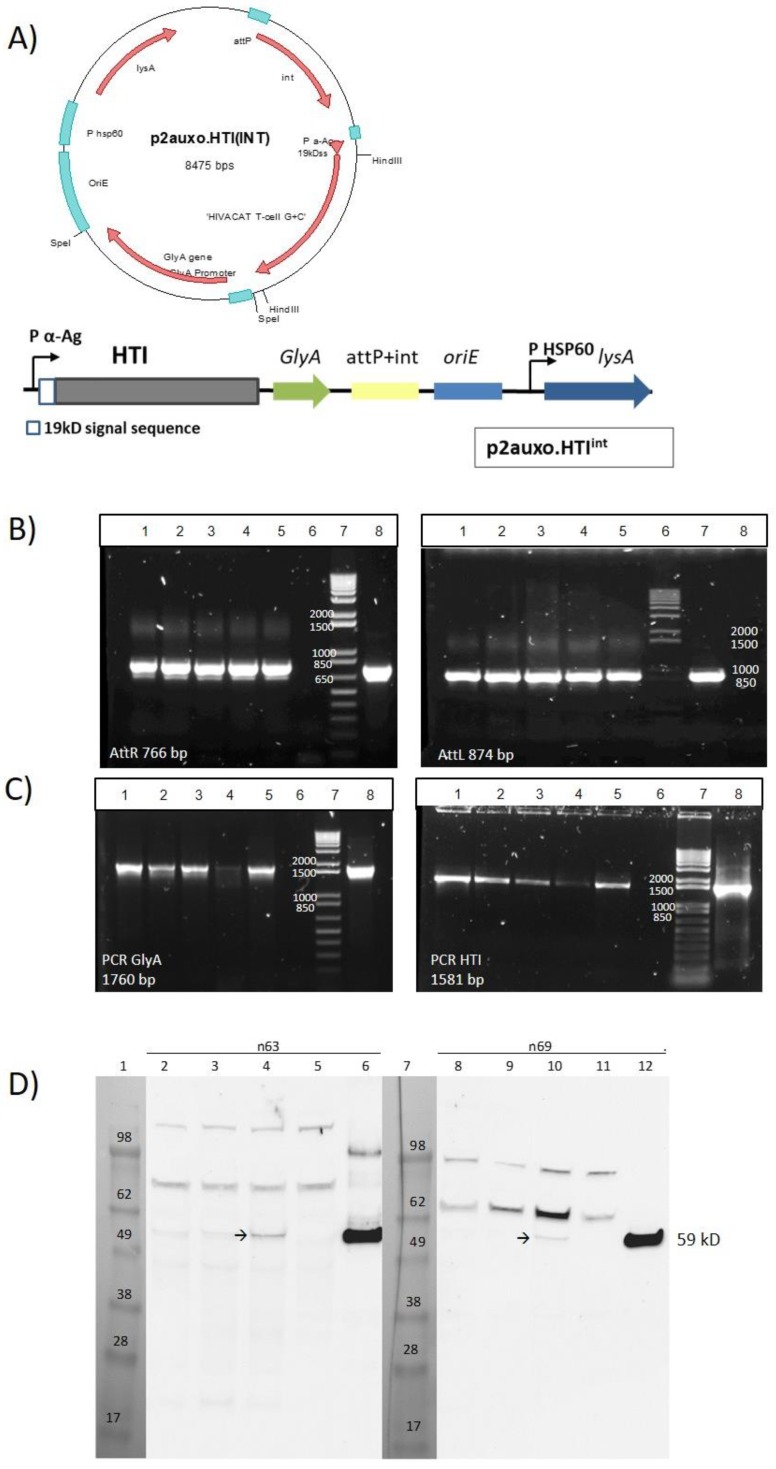
Construction of the recombinant Bacillus Calmette-Guérin HIVACAT T-cell immunogen (BCG.HTI^2auxo.int^) strain. (**A**) The HTI synthetic sequence was BCG codon-optimized and fused to the 19-kDa lipoprotein signal sequence and inserted into the integrative p2auxo.HTI^int^ *E. coli*-mycobacterial shuttle plasmid. This vector contains P α-Ag, which is a *Mycobacterium tuberculosis* α-antigen promoter, PHSP60, which is a heat shock protein 60 gene promoter. The *glyA* and *LysA* complementing genes function as an antibiotic-free selection and maintenance system in the auxotrophic strains of *E. coli* M15Δ*glyA* and BCGΔ*Lys*, respectively; (**B**) PCR analysis of recombinant BCG clones transformed with p2auxo.HTI^int^ for integration sites: “*attR*” (left panel; lanes 1–5; clones 1–5; lane 6: BCG.empty^2auxo.int^; lane 7: molecular weight marker; lane 8: p2auxo.HTI^int^) and “*attL”* (right panel; lanes 1–5; clones 1–5; lane 6: molecular weight marker; lane 7: p2auxo.HTI^int^; lane 8: BCG.empty^2auxo.int^), (**C**) PCR analysis using primers specific for *glyA* (left) and HTI (right) on BCG transformed with p2auxo.HTI^int^ lanes 1–5; clones 1–5; lane 6: BCG.empty^2auxo.int^; lane 7: molecular weight marker; lane 8: p2auxo.HTI^int^. (**D**) Western blot of BCG.HTI^2auxo.int^ lysates; lanes 1 and 7: Molecular weight marker; lanes 2 and 8: Master Seed of BCG.HTI^2auxo.int^ clone 1; lanes 3 and 9: Master seed of BCG.HTI^2auxoint^ clone 3; lanes 4 and 10: Working Vaccine Stock of BCG.HTI^2auxo.int^ clone 3; lanes 5 and 11: BCGwt (negative control); lanes 6 and 12: purified recombinant HTI protein. The HTI proteins were detected using the n63 (left) and n69 (right) mAbs directed against the HTI protein (AELIX Therapeutics, Barcelona, Spain) followed by horseradish peroxidase-goat-anti-mouse and enhanced chemiluminescence (ECL) detection.

In the p2auxo.HTI^int^ *E. coli*-mycobacterial shuttle vector, the heterologous open reading protein expression cassette is under the control of the *Mtb* α-antigen promoter, which is a weak promoter that has been shown to enhance protein stability [37]. The open reading frame of the heterologous protein is initiated by the mycobacterial 19-kDa protein signal sequence, which at its 5’ end was fused to the HTI coding sequence. This enables the localization of the newly synthesized HTI polyprotein to the mycobacterial membrane, and subsequently its secretion, to prevent the internal accumulation of the heterologous protein and enhance protein immunogenicity (Figure 1A). The plasmid contains the *E. coli* origin of replication (*oriE*), attachment sites (*attP*), and the integrase (*int*) genes from the mycobacteriophage L5 [38], and integrates as a single copy into the *attB* region on the BCG chromosome. The plasmid also contains the wild-type glycine A-complementing gene (*glyA*) and lysine A-complementing gene (*lysA5*) for vector selection and maintenance in the auxotrophic *E. coli* and BCG strain, respectively [39,40]. The p2auxo.HTI^int^ was obtained following the methodology previously described [33] and transformed into the glycine auxotrophic *E. coli* M15ΔglyA strain and the lysine auxotrophic BCG Pasteur strain (*ΔlysA5*) [41,42]. The positive recombinant *E. coli* colonies were selected through culture on Minimal M9-D agar plates and the BCG.HTI^2auxo.int^ colonies on Middlebrook agar 7H10 medium without lysine supplementation. Integration of the p2auxo.HTI^int^ plasmid DNA into the mycobacterial genome was assessed by PCR analysis of the *attR* and *attL* DNA insertion regions. The BCG.HTI^2auxo.int^ was used as a template, and bands of 766 bp and 874 bp corresponding to the *attR* and *attL* DNA regions were detected (Figure 1B), demonstrating that p2auxo.HTI^int^ had integrated at the *attB* genomic BCG DNA region. Expression of the full-size chimeric 19-kDa signal sequence-HTI protein was confirmed by Western blot analysis of BCG.HTI^2auxo.int^ lysates (Figure 1C). The selected clones were preserved using the seed-lot system. Clone #3 was selected as the candidate, and Master Seed stocks and Working Vaccine Stocks were prepared for further molecular characterization, immunogenicity, and safety testing in mice. 

### 3.2. Genetic Identification and Characterization of BCG.HTI^2auxo.int^

**Figure 2:**
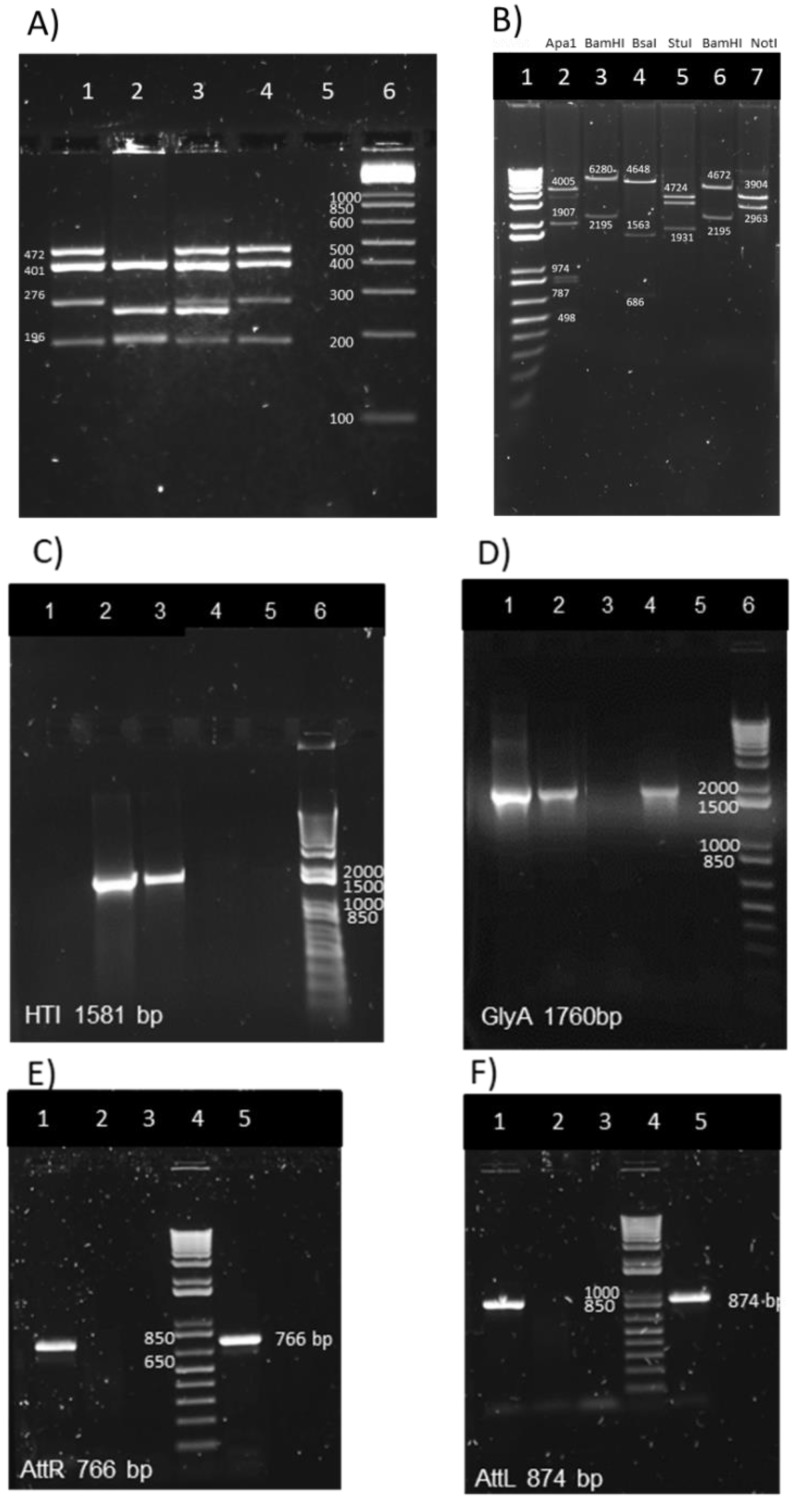
Genetic characterization of the BCG.HTI^2auxo.int^ strain. (**A**) Pasteur substrain identification of BCG.HTI^2auxo.int^ strains by multiplex PCR assay. Lane 1: BCG.HTI^2auxoint^ Working Vaccine Stock giving the bands of 472 bp, 401 bp, 276 bp, and 196 bp; lane 2: BCG Connaught giving the bands of 401 bp, 252 bp, and 196/199 bp; lane 3 BCG Danish with bands of 472 bp, 401 bp, 276 bp, 252 bp, and 196 bp, lane 4: BCG Pasteur with bands of 472 bp, 401 bp, 276 bp, and 196 bp; lane 5: negative control, distilled water; and lane 6: Molecular weight marker. (**B**) Enzymatic restriction analysis of p2auxo.HTI^int^ plasmid DNA purified from transformed *E. coli* M15∆glyA cultures (pre-BCG transformation). Lane 1: molecular weight marker; Lane 2: *Apa1* digestion of p2auxo.HTI.int; Lane 3: *BamHI* digestion of p2auxo.HTI^int^; Lane 4: *Bsal* digestion of p2auxo.HTI^int^; Lane 5: *StuI* digestion of p2auxo.HTI.int; Lane 6: *BamHI* digestion of p2auxo.empty^int^; Lane 7: *NotI* digestion of p2auxo.empty^int^. (**C**) PCR detection of the HTI gene in the BCG.HTI^2auxo.int^ WVS; lane 1: H_2_O; lane 2: p2auxo.HTI^int^ plasmid; lane 3: working vaccine stock of BCG.HTI^2auxo.int^; lane 4: BCG wt; lane 5: BCG transformed with empty 2auxo.int plasmid; lane 6: molecular weight marker. **(D)** PCR detection of the *GlyA* gene in the BCG.HTI^2auxo.int^ working vaccine stock; lane 1: p2auxo.HTI^int^ plasmid; lane 2: WVS of BCG.HTI^2auxo.int^; lane 3: BCGwt; lane 4: BCG transformed with empty 2auxo.int plasmid; lane 5: water; lane 6: molecular weight marker. PCR for right integration site, *attR* (**E,F**) left integration site, *attL* in the BCG.HTI^2auxo.int^ WVS; lane 1 WVS of BCG.HTI^2auxo.int^; lane 2: BCGwt; lane 3: distilled water; lane 4: molecular weight marker; lane 5: positive control, BCG.HIVconsv1^2auxo.int^ Working Vaccine Stock (recombinant BCG expressing the HIVconsv1 immunogen [36,43]).

To confirm that the identity of the BCG.HTI^2auxo.int^ vaccine strains corresponded to the BCG Pasteur substrain, a multiplex PCR-based method was performed to analyze the BCG regions of difference such as RD1, 2, 8, 14, and 16 and the SenX3-RegX3 regions [35]. Different multiplex profiles obtained by this method allow the differentiation of BCG substrains. A PCR product of 196-bp length was generated using the primers ET1-3, indicating deletions of the RD1 region that were only found in BCG strains, not in the *Mycobacterium bovis* or *Mycobacterium tuberculosis* strains. The presence of the RD8 and RD16 regions was confirmed in the BCG.HTI^2auxo.int^ and the BCG Pasteur substrain, which generated products of 472 bp and 401 bp, respectively. Products of 276 bp representing the SenX3-RegX3 region were also found. The molecular patterns of the BCG Danish, BCG Connaught, and BCG Pasteur substrains (Figure 2A) were consistent with previously published patterns [35]. 

PCR and enzymatic restriction analysis were performed to characterize the p2auxo.HTI^int^ plasmid DNA. Following transformation of the *E. coli* M15 Δ*glyA* strain, plasmid DNA was purified, and the enzymatic restriction analysis revealed results that were consistent with the predicted enzymatic pattern (Figure 2B): *ApaI* digestion (Lane 2; bands of 4005 bp, 1907 bp, 974 bp, 787 bp, and 498 bp), *BamHI* digestion (Lane 3; bands of 6280 bp and 2195 bp), *Bsal* digestion (lane 4; bands of 4648 bp, 1563 bp, and 686 bp), *Stul* digestion (Lane 5; bands of 4672 bp and 1931 bp). The empty plasmid p2auxo.∅^INT^ restriction enzyme pattern was also consistent with the expected band pattern: *BamHI* (lane 6; 4672 bp and 2195 bp) and *Notl* (lane 7; bands of 3904 bp and 2963 bp). Next, we performed PCR analysis using specific primers for the HTI and E. coli *glyA* DNA coding sequences using the BCG.HTI^2auxo.int^ Working Vaccine Stock as template. Bands of 1581 bp and 1760 bp corresponding to the expected size of HTI (Figure 2C) and the E. coli *glyA* DNA sequence (Figure 2D) were detected. Furthermore, PCR analysis using specific primers designed for 3’ and 5’ HTI ends was employed to confirm integration of the p2auxo.HTI^int^ plasmid DNA into the parental BCGΔ*lysA* strain genome. The BCG.HTI^2auxo.int^ Working Vaccine Stock was used as a template. Bands of 766 bp and 874 bp corresponding to the respective *attR* (Figure 2E) and *attL* (Figure 2F) attachment sites were detected in Working Vaccine Stocks, but not in BCG wt. The HTI DNA coding sequence was detected by PCR in the BCG.HTI^2auxo.int^ after 35 bacterial generations (Appendix Figure A1).

### 3.3. Phenotypic Characterization of BCG.HTI^2auxo.int^

**Figure 3:**
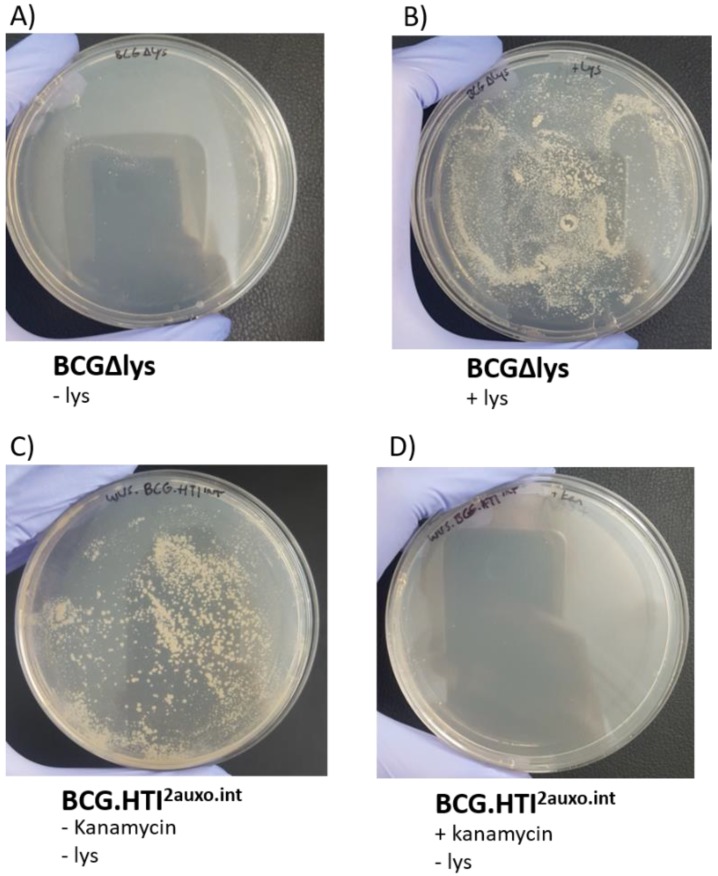
Phenotypic characterization of the BCG.HTI^2auxo.int^ vaccine strain. Phenotype of lysine auxotrophy, lysine complementation, and kanamycin resistance. The BCG lysine auxotroph prior to transformation with p2auxo.HTI^int^ was cultured on non-lysine supplemented 7H10 (**A**) or on lysine supplemented 7H10 (**B**). The BCG.HTI^2auxo.int^ WVS was cultured on 7H10 without lysine or kanamycin supplementation or without lysine but with kanamycin supplementation (**C**,**D**, respectively).

To preserve plasmid stability, both in vivo and in vitro, as well as to prevent potential genetic rearrangements, several factors should be considered when constructing mycobacterium-based vaccine candidates. We previously demonstrated that the use of weak promoters (*Mycobacteria spp*. α-antigen promoter) and use of the BCG lysine auxotrophy-complementation system prevent the disruption of gene expression due to genetic rearrangements [33,37]. The lysine auxotrophic BCG strain was used in combination with lysine gene complementation as an antibiotic-free plasmid selection and maintenance system. To phenotypically assess the stability and demonstrate the lack of antibiotic resistance of this system, the BCG.HTI^2auxo.int^ strain was cultured on non-lysine-supplemented agar with and without kanamycin. In line with previous findings, the untransformed lysine auxotrophic BCG strain failed to grow without the presence of lysine and grew upon supplementation with lysine (Figure 3A,B). The BCG.HTI^2auxo.int^ strain, on the other hand, grew on non-lysine supplementation (Figure 3C), and did not grow on agar plates containing kanamycin (Figure 3D). 

### 3.4. The BCG.HTI^2auxo.int^ prime-ChAdOx1.HTI Boost Regimen Elicits HIV-1-Specific T-cell Responses 

**Figure 4:**
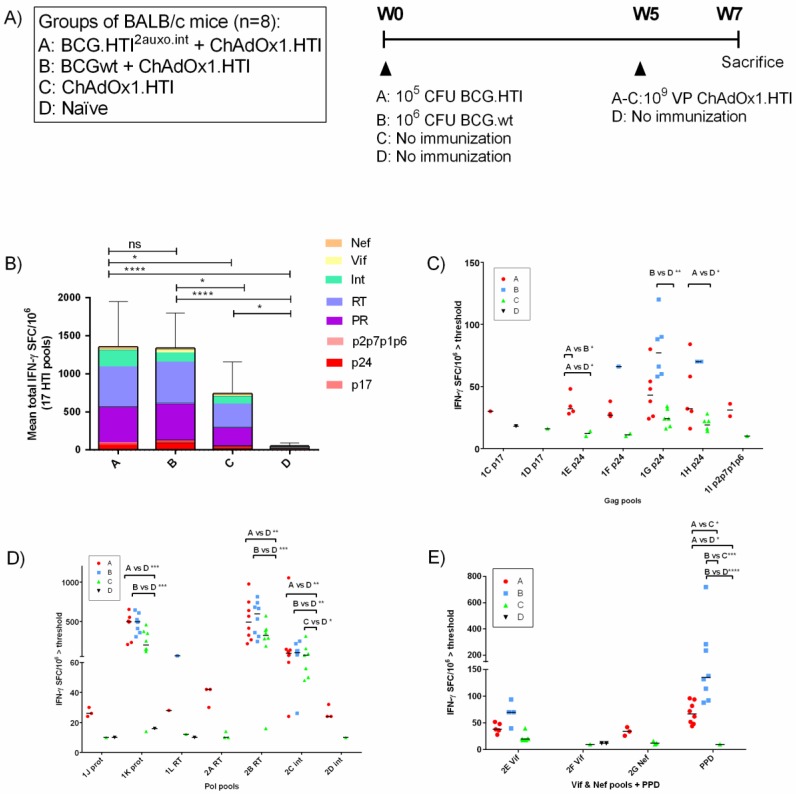
Induction of HIV-1 specific T-cell responses by the BCG.HTI^2auxo.int^ + chimpanzee adenovirus HIVACAT T-cell immunogen (ChAdOx1.HTI) prime-boost regimen in BALB/c mice. Adult mice (seven weeks old, *n* = 8/group) were immunized with either 10^5^ cfu of BCG.HTI^2auxo.int^ (id) and boosted with ChAdOx1.HTI (10^9^ vp, im) after five weeks (group A), or with 10^6^ BCG.wt (id) and boosted with ChAdOx1.HTI (10^9^ vp, im) after five weeks (group B), or only immunized with ChAdOx1.HTI (10^9^ vp, im) at week five (group C), or left unimmunized (group D). Groups and the immunization schedule are shown in (**A**). Two weeks post-boost, mice were sacrificed, and splenocytes were isolated for enzyme-linked immune absorbent spot (ELISpot) analysis. (**B**) The total magnitude of HIV-1 specific SFCs/10^6^ splenocytes was calculated as sums of the SFCs elicited by the 17 HTI peptide pools, the color-coding represents the HIV-1 gene location of the pools. Data are presented as group means and error bars represent the standard deviation of the total sum of SFC/10^6^ splenocytes. Statistics were performed using parametric one-way ANOVA. (C-E) HIV-1 and tuberculosis (TB)-specific T-cell responses interferon-γ (IFN-γ spot-forming cells SFC/10^6^ in response to HTI-derived peptide pools representing HIV-1 Gag (**C**), HIV-1 Pol (**D**), and Nef, Vif, and tuberculin purified protein derivative (PPD) (**E**). The data are presented as medians of group responses above the threshold. Statistics were performed using the non-parametric Kruskal–Wallis test adjusted for multiple comparisons, *p < 0.05, **p < 0.01, ***p <0.001 and ****p < 0.0001.

In order to assess the enhancement of cellular immune responses provided by a prime vaccination with BCG.HTI^2auxo.int^, adult mice (seven-week-old, *n* = 8/group) were immunized with either 10^5^ cfu of BCG.HTI^2auxo.int^ (id) and boosted with ChAdOx1.HTI 10^9^ viral particles (vp) delivered intramuscularly (im) after five weeks (group A), or with 10^6^ BCGwt (id) and boosted with ChAdOx1.HTI (10^9^ vp, im) after five weeks (group B), or only immunized with ChAdOx1.HTI (10^9^ vp, im) at week five (group C), or left unimmunized (group D). The groups and immunization regimens are illustrated in Figure 4A. A group primed with BCGwt was included to allow comparison of the unspecific adjuvanticity of BCG and the specific priming of BCG expressing HTI. Two weeks post-boost, mice were sacrificed and splenocytes were isolated for an ELISpot analysis of IFN-γ secretion in response to 17 peptide pools spanning the HTI proteome. The total magnitude of IFN-γ secreting cells in response to HTI peptide pools was approximately doubled when priming with BCG.HTI^2auxo.int^ as compared to ChAdOx1.HTI alone; however, the same was observed in BCGwt primed mice (Figure 4B). Overall, priming with BCG.HTI^2auxo.int^ or BCGwt increased responses to peptide pools in the responding mice (Figure 4C–E), although these differences only reached trends when compared with animals only receiving ChAdOx1.HTI. In mice immunized with ChAdOx1.HTI alone, statistically significant differences as compared to naïve mice were only observed in response to one pool representing integrase (int) (pool 2C, Figure 4D). Priming with BCG.HTI^2auxo.int^ induced significantly higher responses as compared to naïve mice in response to five HTI-derived pools (Figure 4: 1E p24; *p* = 0.0469, 1H p24; *p* = 0.048, 1K prot; *p* = 0.0004, 2B RT; *p* = 0.0011, and 2C int; *p* = 0.0038). A similar number was observed for mice primed with BCGwt (Figure 4: 1G p24; *p* = 0.0016, 1K prot; *p* = 0.0002, 2B RT; *p* = 0.0004, and 2C int; *p* = 0.0360). However, the IFN-γ response to pool 1E representing p24 was significantly higher in mice receiving BCG.HTI^2auxo.int^ as compared to those receiving BCGwt (Figure 4C). Both the recombinant and wild-type BCG induced *Mtb*-specific responses (PPD, Figure 4E).

**Figure 5:**
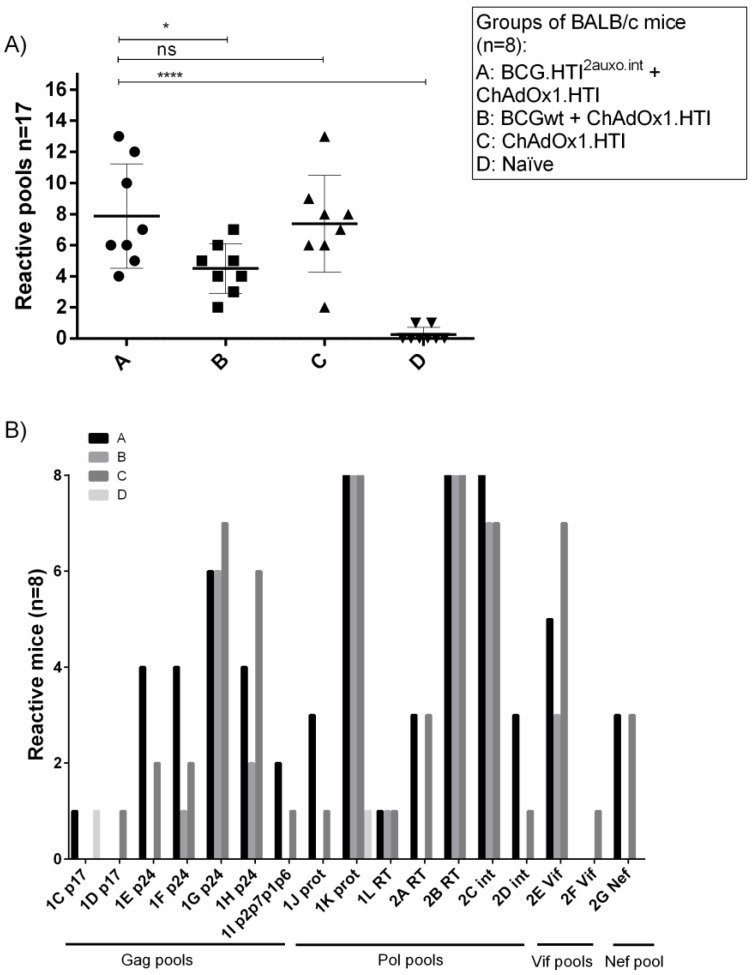
Differential recognition of peptide pools in BCG.HTI^2auxo.int^ + ChAdOx1.HTI immunized BALB/c mice. Adult mice (seven weeks old, *n* = 8/group) were immunized with either 10^5^ cfu of BCG.HTI^2auxo.int^ (id) and boosted with ChAdOx1.HTI (10^9^ vp, im) after five weeks (group A), or with 10^6^ BCG.wt (id) and boosted with ChAdOx1.HTI (10^9^ vp, im) after five weeks (group B), or only immunized with ChAdOx1.HTI (10^9^ vp, im) at week five (group C), or left unimmunized (group D). Two weeks post-boost, mice were sacrificed, splenocytes were isolated for ELISPOT analysis, and the numbers of reactive peptide pools (total n peptide pools =17) were compared for each mouse. (**A**) The number of reactive pools per mouse. (**B**) The number of reactive mice (eight mice per group) in each group according to peptide pool and HIV-1 gene location. Statistics were performed using parametric one-way ANOVA, *p < 0.05, **p < 0.01, ***p < 0.001, ****p < 0.0001.

Interestingly, a comparison of the average number of reactive HTI peptide pools in vaccinated mice revealed that the number of reactive pools in BCGwt primed mice was significantly lower than in those primed with BCG.HTI^2auxo.int^ (Figure 5A, *p* = 0.0262). This indicates a loss of breadth when priming with BCGwt, even though IFN-γ secreting cells in response to HTI peptide pools were similar when compared with BCG.HTI^2auxo.int^ primed mice. No differences between BCG.HTI^2auxo.int^ primed mice as compared to mice only receiving ChAdOx1.HTI (Figure 5A) were observed regarding the number of pools recognized per mouse, although the mean was slightly increased from 7.3 to 7.8 when priming with BCG.HTI^2auxo.int^. The highest number of recognized peptide pools was 13 in both groups. Interestingly, certain mice of the BCG.HTI^2auxo.int^ + ChAdOx1.HTI immunized group reacted to pools less frequently recognized by other groups (Figure 5B: 1E p24, 1F p24, 1J prot, 2D int). On the other hand, lower numbers of mice recognized certain peptide pools (Figure 5B: 1G p24, 1H p24, 2E Vif) when compared to mice only immunized with ChAdOx1.HTI.

### 3.5. The BCG.HTI^2auxo.int^ + ChAdOx1.HTI Prime-Boost Regimen Is Well Tolerated

**Figure 6:**
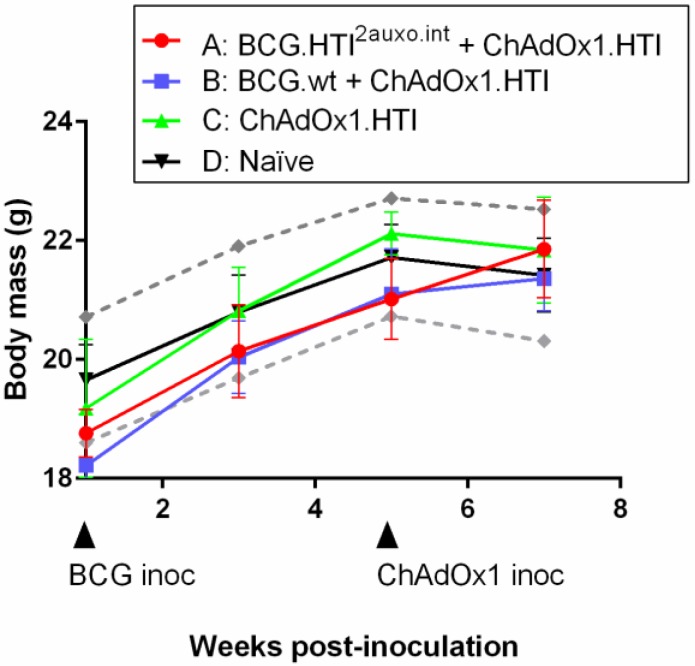
Safety of the BCG.HTI^2auxo.int^ and ChAdOx1.HTI prime-boost regimen in BALB/c mice. Mice in groups of five (female, seven weeks old) were immunized i.d. with 10^5^ colony-forming units (CFU) of BCG.HTI^2auxo.int^ or 10^6^ CFU BCGwt and boosted with 10^9^ VP of ChAdOx1.HTI. Body weights were recorded regularly, and the mean for each group of mice is shown as mean ± SD (n = 5). Data from naive mice are presented as mean ± 2 SD (n = 5) (dashed grey lines).

Five adult mice per group were either left unimmunized or received 10^6^ colony-forming units (cfu) of BCG wt or a total of 10^5^ cfu of BCG.HTI^2auxo.int^ intradermally, and five weeks later were boosted with 10^9^ vp of ChAdOx1.HTI and their body mass was monitored regularly over time (Figure 6). The body mass curve corresponded to those of the provider (Envigo, Huntington, UK), and there were no statistically significant differences observed between the vaccine recipients and controls at any time point tested (Figure 6). Mice were monitored weekly for signs of malaise. No vaccine-related deaths, no local adverse events, and no associated systemic reactions were observed.

## 4. Discussion

Despite the exploration and implementation of numerous HIV-1 prevention strategies, 1.8 million new HIV infections occurred in 2017 [44]. There is an urgent need for the development of an effective, safe, and affordable HIV-1 vaccine. We have constructed an rBCG vaccine candidate expressing the HIVACAT T-cell immunogen, HTI, using the integrative antibiotic-resistance free *E. coli*-mycobacterial shuttle vector, p2auxo.int. The HTI immunogen was designed to target T-cell responses to the most beneficial T-cell targets and the most vulnerable sites of HIV-1. When delivered using DNA and MVA vectors, it has been shown to be capable of inducing broadly and evenly distributed immune responses of high magnitude in mice and monkeys [7]. We have previously demonstrated the in vitro and in vivo stability of the integrative plasmid p2auxo.int for expressing HIV-1 immunogens in BCG [33,43]. Here, we produced the BCG.HTI^2auxo.int^ vaccine candidates under Good Laboratory Practice compatible conditions, and characterized them genotypically and phenotypically, confirming the presence of the HTI gene and protein in the lysates of working vaccine stocks. Furthermore, we demonstrate that the BCG.HTI^2auxo.int^ vaccine in combination with ChAdOx1.HTI induced TB and HIV-1-specific IFN-γ-producing T-cell responses in adult BALB/c mice. The vaccination regimen was well tolerated during the follow-up period, although a longer safety assessment will be necessary, as symptoms related to a lack of attenuation of mycobacteria could take at least 50 days to emerge and impact body mass in the mouse model [45]. 

BCG is a remarkable live vaccine vehicle due to its capability of delivering antigens to APCs enabling the development of antigen-specific cell-mediated immune responses [24]. Mycobacterial antigens have been shown to be presented to T-cells by non-classical antigen presentation molecules such as CD1a/b/c, Major histocompatibility complex class I-related gene protein (MR1), and Human leukocyte antigen-E (HLA-E). The latter is of specific interest in the context of HIV-1 vaccine development due to the resistance of HLA-E to downregulation by the HIV-1 Nef during infection [46], as well as displaying a low level of allelic variation, with only forty three existing variants as opposed to the thousands of classical HLA class I molecules [47]. Furthermore, BCG-immunized humans elicited HLA-E restricted CD8^+^ T-cell responses to *Mtb* peptides, which display cytotoxic as well as immunoregulatory activities [48]. One of the most successful HIV-related vaccine trials in animal models has been a Cytomegalovirus (CMV) vectored vaccine against SIV. This vaccine was able to establish persistent, SIV-specific effector memory T-cell responses in rhesus macaques and control pathogenic SIV infection following mucosal challenge [49]. CMV infection is known to upregulate HLA-E expression in humans, and the vaccine regimen in the monkeys induced strong Mamu-E restricted T-cell responses [50]. It is still unknown if BCG can elicit a similar immune response to a heterologous antigen when used as a vaccine vector in humans. However, BCG administered as an oral adjuvant along with inactivated simian immunodeficiency virus (SIV)mac239 particles in Chinese macaques was shown to confer protection to a high-dose SIVmac239 challenge [51]. The protection was attributed to non-cytolytic Major histocompatibility complex (MHC) Ib/E-restricted CD8^+^ T-regulatory cells that suppressed the activation of SIV-positive CD4^+^ T-lymphocytes.

As a vector, rBCG shares several traits with plasmid DNA. Both are often used as priming agents in combination with a virally vectored boost [8,24]. A rBCG–DNA prime-boost regimen showed less immunogenicity when explored using the HIVA immunogen, as compared to boosting with viral vectors [28]. On the other hand, a combination regimen of rBCG and DNA expressing HIVA was shown to confer protection in a pathogenic vaccinia–HIVA surrogate challenge model [39]. However, little is known about the advantages for specific immunity to heterologous immunogens upon combining rBCG, DNA, and viral vectors.

Recombinant BCG delivered on its own induces weak transgene-specific immune responses that are difficult to measure. Thus, we have assessed the enhancement of HIV-1 specific cellular immune responses by a prime vaccination with BCG.HTI^2auxo.int^ when delivered with ChAdOx1.HTI in BALB/c mice. The magnitude of the total T-cell response was significantly higher in BCG.HTI^2auxo.int^ primed mice as compared to mice receiving ChAdOx1.HTI alone. A similar magnitude was observed in BCGwt primed mice (Figure 4). The IFN-γ secretion in response to the individual HTI peptide pools was higher in all the assessed pools, although the differences did not reach statistical significance between mice receiving BCG prime and those not. The evident priming effect, even by BCGwt, is in line with the ability of BCG derivatives to act as potent adjuvants for subsequent boosting vaccines. It is known that immunization with BCG not expressing any transgene often can lead to higher vaccine-specific responses when delivered as a prime/adjuvant in combination with a virally vectored vaccine [39,52]. Components of BCG have also been used as an adjuvant for an HIV-1 DNA vaccine [53]. 

Interestingly, both BCG.HTI^2auxo.int^ primed mice and mice receiving ChAdOx1.HTI alone responded to an average of seven to eight peptide pools, whereas mice primed with BCGwt alone only responded to an average of 4.5 peptide pools. This together suggests that priming with BCG.HTI^2auxo.int^ enhances the HTI-specific immune response when delivered with ChAdOx1.HTI, while maintaining the breadth of the response. Priming with BCGwt appeared to boost the overall magnitude of the response, but ultimately directing responses to fewer peptide pools. The differences in the breadth of immune responses between the BCG.HTI^2auxo.int^ and BCGwt could possibly be related to the strong adjuvant properties of BCG. A possible explanation is its capability of inducing trained immunity. This involves enhanced monocyte function and Natural killer (NK) cell function as reviewed by van der Meer et al. [54]. Macrophages, monocytes, and natural killer cells display enhanced responsiveness following a second encounter with a pathogen. Enhanced monocyte function has been demonstrated in humans three months after BCG vaccination along with increased cytokine production, as well as CD11b and toll-like receptor 4 (TLR-4) expression [17]. This effect could be observed up to one year following immunization [55]. The unspecific immune activation could perhaps be involved in mechanisms enhancing the production of cytokines such as IFN-γ in T-cells, which leads to higher responses in the epitopes, which are more dominant. It is notable that the HTI was designed to avoid useless immunodominant epitopes in humans, but nevertheless, it is inevitable that there will be a hierarchy of epitopes in mice in which cellular immune responses are elicited against. Thus, the higher breadth observed following priming with the BCG.HTI^2auxo.int^ could be related to the priming of responses that are specifically related to the transgene expressed (HTI), whereas the general increase in IFN-γ induced by BCGwt could be related to unspecific or adjuvanticity-related effects on the immune system rather than the specific priming of immune responses directed toward HTI. Previously, C57BL/6 mice immunized with DNA.HTI alone were shown to respond to two to six HTI peptide pools, and following an immunization schedule consisting of three DNA.HTI prime immunizations and one MVA.HTI immunization delivered at three-week intervals, this number was increased to six to 11 peptide pools [7]. Delivering a combination of BCG.HTI^2auxo.int^, DNA, and a viral vector expressing HTI could present a strategy for increasing the number of recognized pools further. 

Correlates of protection for HIV-1 vaccines have been discussed following the RV144 trial, such as the IgG Ab response to the variable regions 1 and 2 (V1V2) loops being associated with a reduction in HIV-1 acquisition [56]. However, the translatability of data obtained in small animal models is limited, and pre-clinical design and the evaluation of HIV-1 vaccines remains a challenge. In humans, T-cell responses have also been associated with protection or decreased viral loads following infection. For instance, in the RV144 trial, CD4^+^ T-cells secreting IL-2, TNF-α, IFN-γ, IL-4, and CD154 in response to HIV-1 envelope peptides were associated with lower infection rates in vaccine recipients [57]. 

## 5. Conclusions

We here demonstrate that priming with BCG.HTI^2auxo.int^ significantly increased HTI-specific T-cell responses. Although priming with BCGwt led to a similar enhancement of the magnitude of the response, fewer peptide pools were recognized in BCGwt primed animals. The ability of the BCG.HTI^2auxo.int^ priming immunization to increase T-cell immune responses when combined with ChAdOx1.HTI, while maintaining the breadth of the response, strengthens its applicability as a priming vaccine for the development of an efficacious HIV-1 vaccine. Finally, we demonstrate that in some mice, priming with BCG.HTI^2auxo.int^ can alter the immunodominance profile of the vaccine-induced T-cell response. Further assessments and characterization of the T-cell response by intracellular cytokine staining would provide a more thorough overview of the vaccine-induced immune response and of potential functional differences, depending on the different vaccination regimen. 

## Figures and Tables

**Figure A1:**
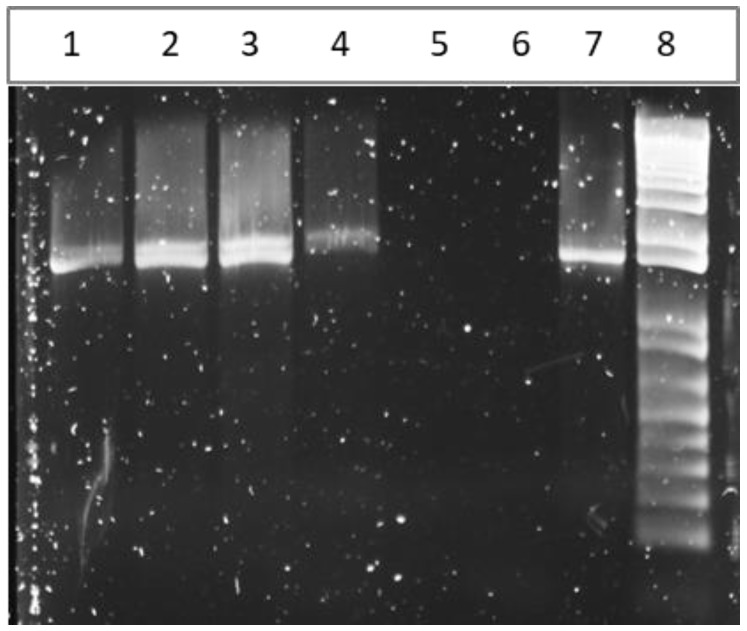
In vitro stability of BCG.HTI^2auxoint^. *In vitro* stability was assessed by PCR detection of the HTI gene in the BCG.HTI^2auxo.int^ master seeds after five weekly subcultures with and without added lysine; lane 1; BCG.HTI^2auxo.int^ clone 1; lane 2: BCG.HTI^2auxo.int^ clone 3; lane 3: BCG.HTI^2auxo.int^ clone 1 cultured in lysine supplemented medium; lane 4: BCG.HTI^2auxo.int^ clone 3 cultured in lysine supplemented medium; lane 5: BCG.Ø^2auxo.int^; lane 6: distilled water; lane 7: p2auxo.HTI.int plasmid; lane 8: molecular weight marker.
